# Correlation of the plasma sphingoid base profile with results from oral glucose tolerance tests in gestational diabetes mellitus

**DOI:** 10.17179/excli2017-171

**Published:** 2017-04-18

**Authors:** Abad Khan, Thorsten Hornemann

**Affiliations:** 1 Institute for Clinical Chemistry, University Hospital Zurich, Ramistrasse 100, 8091 Zurich, Switzerland; 2 Department of Pharmacy, University of Swabi, Khyber Pakhtunkhwa, Pakistan

**Keywords:** gestational diabetes, oral glucose tolerance test, basal glucose, area under the curve, correlation, sphingoid-bases

## Abstract

Oral glucose tolerance test (OGTT) is usually insufficient to accurately predict the risk for type 2 diabetes mellitus (T2DM), it is therefore necessary to identify an additional biomarker that would most likely improve the accuracy of OGTT. The current OGTT was performed in 53 volunteers after ingestion of 75 g glucose in 250 ml water to each volunteer. Similarly the sphingoid base profile of these volunteers was explored using liquid-chromatography linked with mass spectrometer (LC-MS) and correlated with the different time-points glucose values of OGTT as well as with total area under the curve (tAUC), incremental area under the curve (iAUC), and positive incremental area under the curve (pAUC). The findings showed that 1-deoxysphinganine (1-deoxySA) was significantly positively correlated with the 1-hour, 2-hour, and 3-hour plasma glucose level as well as with total, incremental, and positive incremental AUC while 1-deoxysphingosine (1-deoxySO) was correlated only with 1-hour, 2-hour glucose levels and tAUC of OGTT. The C18SAdiene was negatively correlated with all-time points glucose values and AUCs followed by negative correlation of C18SO, C16SO and C17SO with 2-hour glucose and tAUC of OGTT. The ratios of 1-deoxySA and 1-deoxySO with respect to C18SAdiene have shown significant correlation with 2-hour and AUCs. These ratios were higher in subjects with gestational diabetes in comparison with normal subjects. These findings underlined that 1-deoxysphingolipids (1-deoxySLs) and their ratios with C18SAdiene could be significantly correlated with the glucose load of OGTT and might be used as predictive biomarkers along with OGTT for the risk assessment of diabetes.

## Introduction

Gestational diabetes mellitus (GDM) is defined as glucose intolerance of variable severity with onset or first recognition during pregnancy (Schmidt et al., 2001[18]). GDM or impaired glucose tolerance (IGT) that is first diagnosed during pregnancy affects ~14 % of pregnancies and is also a risk factor for type 2 diabetes in the mother. However screening of GDM may be applied for the identification of mothers at higher risk for type 2 diabetes and intervention in subjects with IGT has been revealed to delay the onset of type 2 diabetes (Kim et al., 2002[11]). The currently existing methodologies for the diagnosis of prediabetes such as glycated hemoglobin (HbA1c) and fasting glucose (FG) are inadequate for the detection of individuals at risk as HbA1c may be altered in anemia or renal failure or remain normal in mild dysglycemia (Buysschaert et al., 2015[5]). The OGTT is a useful tool to provoke a metabolic challenge not only for diagnostic purposes but also for scientific purposes to monitor the pharmacokinetic of glucose (Abdul-Ghani et al., 2009[1]). Since the 2-hour OGTT is considered as gold standard method for the diagnosis of type 2 diabetes as it is most commonly used in clinical practice and research for the identification of people with normal or IGT as well as patients with type 2 diabetes (Andrikopoulos et al., 2008[3]). In OGTT blood glucose is usually measured before (after an overnight 10-16 hour fasting) and at 2 or 3 hours after ingestion of 75 g glucose dissolved in 200-250 ml of water. The 2-hours glucose values are then compared with the standard WHO values to know about normal, impaired glucose and diabetes (Kjøllesdal et al., 2014[12]). According to WHO recommendations diabetes can be diagnosed either on elevated fasting blood glucose level (≥ 7 mM) or on 2-hour plasma glucose level (≥ 11 mM) of OGTT after ingestion of 75 g glucose solution preceded by an overnight fasting (WHO, 2006[25]). The WHO criteria based on blood glucose level measured at 2-hour of OGTT is glucose value ≥ 7.8 mmol/l (140 mg/dl) but < 11.1 mmol/l (200 mg/dl) considered as IGT while values ≥ 11.1 mmol/l (200 mg/dl) as indicatives of diabetes (Zhou et al., 2006[30]). As OGTT gives no idea about the total raise in blood glucose level (Kjøllesdal et al., 2014[12]), the area under the curve (AUC) is usually applied to know about the total raise in blood glucose during an OGTT. For assessment of total blood glucose response vs time during OGTT the total, incremental and positive incremental AUC have been applied in several studies with merits and demerits (Wolever and Jenkins, 1986[26]; Wolever et al., 1991[27]; Allison et al., 1995[2]; Kjøllesdal et al., 2014[12]). The shortcoming of the OGTT is the limitation of evaluation only one or few metabolic factors simultaneously. Moreover the conventional risk factors like impaired fasting glucose (IFG) as well as impaired glucose tolerance (IGT) usually fail to predict 50 % of the patients prone to develop T2DM (Othman et al., 2015[16]). However the application of another metabolic biomarker along with glucose will not only enhance the understanding but also increase the precision and accuracy of diagnosis diabetes as well as other metabolic disorder (Andrikopoulos et al., 2008[3]). Metabolomics' approaches have been applied for the identification of novel biomarkers as these can simultaneously quantify metabolites that are products of different metabolic pathways. The identification of such biomarkers, the concentrations of which are altered in diabetes and prediabetes, may be helpful in explaining pathways that are involved in diabetes. These biomarkers may be applied for the detection of earlier and more reliable dysfunction of glucose metabolism than other existing clinical markers of diabetes (Buysschaert et al., 2015[5]). In recent years several metabolites such as amino acids, phospholipids, and sphingolipids are becoming candidates of biomarkers for the prediction of future diabetes (Wang et al., 2011[22]; Wang‐Sattler et al., 2012[23]; Floegel et al., 2013[9]; Othman et al., 2015[16]). 

Sphingoid bases are the shared structural element of all sphingolipids and are typically comprised of backbones 2-amino, 1, 3-diols synthesized from serine and palmitoyl-coenzyme A (palmitoyl-CoA) by the enzyme serine palmitoyltransferase (SPT). However, SPT can also metabolize other acyl-CoAs besides palmitoyl-CoA to produce atypical sphingolipids having atypical chain length in the range of 12 to 26 carbons. In certain conditions many organisms including mammals can also utilize _L_-alanine and glycine instead of _L_-serine to produce 1-deoxy sphingolipids (1-deoxySLs) that are made of 1-deoxysphingoid bases (DSBs) (Othman et al., 2015[16]). A low level of 1-deoxySA and 1-deoxySO are found in plasma of healthy individuals under normal physiological conditions (Wei et al., 2014[24]), however in pathophysiological conditions these are produced in higher amount (Zuellig et al., 2014[31]). Elevated 1-deoxySLs levels have been found in T2DM (Dohrn et al., 2015[7]), metabolic syndrome (MetS) (Wei et al., 2014[24]), impaired fasting glucose (IFG) (Wei et al., 2014[24]), non-alcoholic steatohepatitis (Gorden et al., 2015[10]), and defects in serine biosynthesis (Esaki et al., 2015[8]), and can be predicted for other conditions where metabolic changes or diet alter the amounts (Merrill and Carman, 2015[13]). It is therefore assumed that 1-deoxySLs can be used as predictive biomarkers for the diagnosis of these diseases. This study is designed to link OGTT to various sphingolipids metabolomics to explore novel metabolic biomarkers and pathways which are affected by the glucose metabolism, thereby opening new perceptions to study the physiological behavior of the body on glucose metabolism. The correlation studies of these sphingoid bases with fasting, 1-hour, 2-hour, and 3-hour glucose levels and area under the curve (AUC) were carried out in order to find out the relationship of these sphingolipids with OGTT. 

The aim of this study is to explore the correlation of sphingoid bases with different glucose levels of OGTT and tAUC, iAUC, and pAUC in order to show their relationship with glucose load and discover new metabolic biomarkers and pathways that are influenced by glucose metabolism, thereby opening new perspectives in the study of the physiological reaction of the body on glucose ingestion. Moreover to investigate whether the 1-deoxysphingoid bases can be used as potential biomarkers for the diagnosis of GDM and prediction of risk for T2DM during OGTT in order to improve further the accuracy and precision of OGTT. 

## Materials and Methods

### Subjects

The study was conducted at Department of Clinical Chemistry, University Hospital Zurich. The study was approved by the ethical committee of the institute. Total fifty nine (N=59) volunteers participated in this study after their complete medical history, screening tests and informed written consent in accordance with the guidelines of the hospital for human experimentation and Helsinki Declaration. 

### Oral Glucose Tolerance Test

The subjects reported to the laboratory in the morning after an overnight fasting. All subjects were abstained from food or any other drink except water for 12-16 hours. The fasting blood samples were taken after inserting a catheter into the prominent vein of forearm. A 75 g standard glucose solution was given to the volunteers at 0 time-points. Plasma samples were collected at 1-hour, 2-hour, and 3-hour time intervals from all the volunteers. These plasma samples were then analyzed for glucose using standard biochemical protocols. After the data collection the total, incremental, and positive incremental AUC were measured using the trapezoidal method. 

### Sphingolipid analysis

The different sphingoid bases such as C16-sphingosine (C16SO), C16-sphinganine (C16SA), C17-sphingosine (C17SO), C18-sphingosine (C18SO), C18-sphinganine (C18SA), C18SA-diene, C19-sphingosine (C19SO), and the 1-deoxy-sphingoid bases i.e., 1-deoxy-sphingosine (1-deoxySO) and 1-deoxy-sphinganine **(**1-deoxySA) were analyzed by liquid-chromatography mass spectrometry (LC-MS) in plasma samples of these volunteers. The sphingoid base profile was analyzed by the method reported by Othman et al. (2015[16]). The sphingoid-bases such as C16SO, C16SA, C17SO, C18SO, C18SA, C18SA-diene, C19SO, and DSBs including 1-deoxySO and 1-deoxSA were separated on a C18 column (120 ^º^A, 5 mm, 125 × 2 mm; Uptispere; Interchim, Montlucon, France) using TSQ Quantum Ultra Mass Spectrometer (Thermo, Reinach, Basel-Landschaft, Switzerland) after extraction and hydrolysis as reported earlier (Othman et al., 2012[15], 2015[14]). Isotope-labelled d7-sphingosine (D7SO) and d7-sphinganine (D7SA) (200 pmol; Avanti Polar Lipids, Alabaster, AL) were used as internal standard. The plasma values of these sphingoid bases were quantified.

### Statistical analysis 

Data are represented as mean ± SEM. Statistical correlation studies were carried out using SPSS Statistics 22 (IBM, Zurich, Switzerland) and GraphPad Prism 5 (GraphPad Software, Inc., San Diego, CA). Pearson's correlation coefficient was calculated for the relationship between tAUC, iAUC, and pAUC and glucose values measured at 0, 1-hour, 2-hour, and 3-hour time interval during OGTT. The total glucose response vs time was evaluated by area under the curve (AUC). For measuring the total glucose response vs time during an OGTT we applied the trapezoidal rule (Purves, 1992[17]) to calculate total, incremental, and positive incremental AUC. The tAUC depends on basal glucose value while the iAUC and pAUC are not related to basal glucose value. Since in incremental AUC the baseline values are subtracted from the subsequent values therefore a negative value will be obtained if any of the post baseline values are lower than the baseline value which controverts the concept of area in the physical sense (Allison et al., 1995[2]). Similarly during calculating the positive incremental AUC only the values above the baseline values are considered and those below the baseline are ignored (Wolever and Jenkins, 1986[26]; Wolever et al., 1991[27]). This also does not give the true picture of the data as it throws away much of the variance in any reading below the baseline. So the total AUC represents the actual determination of total area under the curve and also minimizes the concerns with the other methods. Since the tAUC is independent of the ever-changing baseline glucose or insulin level therefore it might be the preferred method for the evaluation of a response during OGTT. The Pearson's correlation coefficients were calculated for the relationship between sphingoid bases and glucose levels measured at 0, 1-hour, 2-hour, and 3-hour time interval during OGTT as well as for the relationship between sphingoid bases and total, incremental, and positive incremental AUC. Similarly the ratios of 1-deoxySA and 1-deoxySO with respect to C18SAdiene were correlated with 2-h glucose values and tAUC of OGTT. The level of significance was preset at 5 % (P ≤ 0.05). 

## Results

The descriptive statistics of glucose values measured at random, 0, 1, 2, and 3-hour time-points during an OGTT as well as age and plasma sphingoid bases of the under study subjects are given in Table 1(Tab. 1). The values have been presented as minimum, maximum, and mean with their respective standard deviation. The mean age of the participants is 33.50 years. The mean ± SD values for fasting, 1-hour, 2-hour, and 3-hour glucose during OGTT are 3.981 ± 1.70, 8.043 ± 1.945, 6.430 ± 1.832, and 2.817 ± 2.194 mMol/L, respectively. The data are showing greater variation in the glucose values at 1, 2, and 3-hour of OGTT. The values of 1-deoxySA and 1-deoxySO are 0.199 ± 0.119 and 0.140 ± 0.103 mΜ/L, respectively. The total, incremental, and positive incremental AUC were calculated using the trapezoidal rule for the evaluation of the total blood glucose response vs time during OGTT. The respective mean ± SD values of tAUC, iAUC, and pAUC are 18.234 ± 4.146, 7.919 ± 4.767, and 8.769 ± 4.277 mM.h/L as given in Table 1(Tab. 1). 

**Figure 1:**
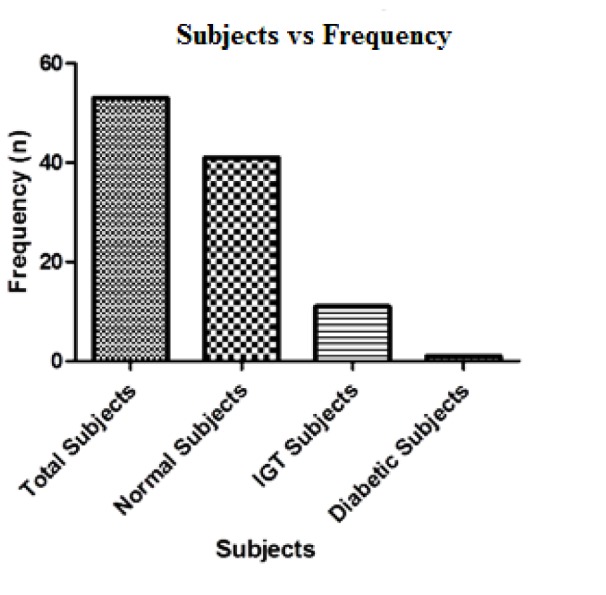
Distribution of subjects as normal (41), IGT (11) and diabetic (1) on WHO criteria based on blood glucose level measured at 2-hour during an Oral Glucose Tolerance Test (OGTT) (glucose value ≥ 7.8 mmol/l (140 mg/dl) but < 11.1 mmol/l (200 mg/dl) considered as IGT while values ≥ 11.1 mmol/l (200 mg/dl) as indicatives of diabetes) (N=53).

Based on the WHO guidelines for 2-hour post load blood glucose values the individuals were screened for IGT and diabetes. It has been found that out of 53 individuals only one has been diagnosed with diabetes as the 2-hour blood glucose value was 11.9 mmol/L (≥ 11.1 mmol/L), 11 (20.75 %) were diagnosed with IGT as their 2-hour blood glucose values were ≥ 7.8 mmol/L, while the remaining 41 (77.36 %) were found normal. The screening results of our study based on WHO 2-hours blood glucose values are graphically represented in Figure 1(Fig. 1).

**Figure 2:**
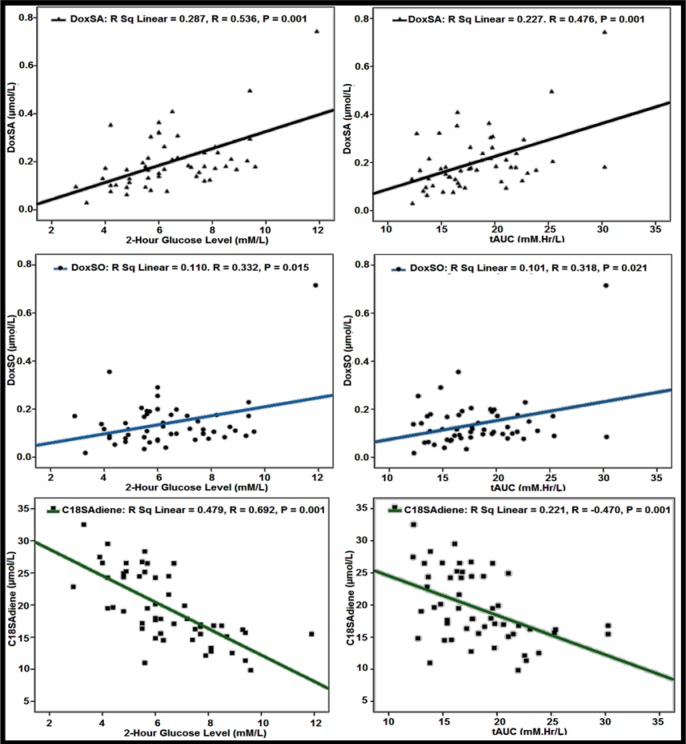
Scatterplots for the relationship between doxSA, doxSO, C18SAdiene and 2-hour blood glucose values and total Area Under the Curve (tAUC), during an Oral Glucose Tolerance Test (OGTT). *Note:* Pearson's correlations' coefficients for doxSA, doxSO, C18SAdiene and 2-hour glucose level of OGTT are (r=0.536; r=0.332 and r= ‒ 0.692) and for total area under the curve (tAUC) of OGTT are (r= 0.476; r= 0.318; and r= ‒0.470) (N=53; p ≤ 0.001).

The Pearson's correlations of various sphingoid bases with age, 0 time (fasting), 1-hour, 2-hour, and 3-hour glucose levels have been carried out to find out the relationship of the sphingoid bases with the different time points glucose level of OGTT as shown in Table 2(Tab. 2). 1-deoxySA was highly correlated with 2-hour glucose level (0.536), followed by 1-hour (0.418) glucose values of OGTT. Similarly 1-deoxySO was best correlated with 2-hour glucose values (0.332), followed by 1-hour glucose values (0.309) of OGTT. The correlations of both of these sphingoid bases with fasting and 3-hour glucose values of OGTT were non-significant. All the other sphingoid bases' correlations were either negative or non-significant. The best negative correlation was shown by C18SAdine with 2-hour glucose values (‒0.692), followed by 1-hour glucose values of OGTT. Similarly the other sphingoid bases that were highly negatively correlated with 2-hour glucose values of OGTT include C18SO (‒0.581), C16SO (‒0.548), and C17SO (‒0.404), respectively. The correlations of C16SA, C18SA, and C19SO were non-significant with the glucose values of OGTT. The Pearson's correlation of the sphingoid bases with tAUC, iAUC, and pAUC was also calculated. The best correlation has been shown for 1-deoxySA with tAUC (0.476), followed by its correlation with pAUC (0.348), and iAUC (0.329), respectively. The correlation of 1-deoxySO with tAUC was significant (0.318), while with pAUC, and iAUC its correlations were non-significant. The C18SAdiene was best negatively correlated with pAUC (‒0.560), followed by iAUC (‒0.530), and tAUC (‒0.470), respectively. Similarly C18SO, C17SO, and C16SO were highly negatively correlated with tAUC, iAUC, and pAUC, while correlations of C16SA, C18SA, and C19SO with tAUC, iAUC, and pAUC were non-significant as shown in Table 2(Tab. 2). The scatter plots of 1-deoxySA, 1-deoxySO and C18SAdiene concentration vs 2-hour glucose levels and tAUC of OGTT are presented in Figure 2(Fig. 2).

**Figure 3:**
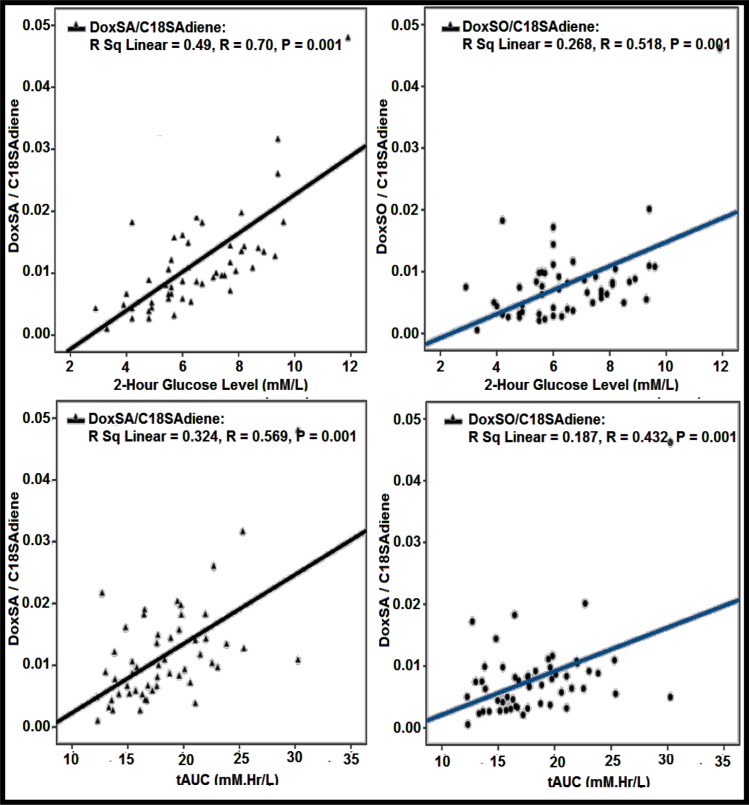
Scatterplots for the relationship between ratios of (1-deoxySA/C18SAdiene) and (1-deoxySO/ C18SAdiene) with 2-hour blood glucose values and total Area under the Curve (tAUC) of an Oral Glucose Tolerance Test (OGTT) (N=53).

The ratios of 1-deoxySA, and 1-deoxySO concentration with respect to C18SAdiene concentration during OGTT were measured. The Pearson's correlation of these ratios with glucose levels and AUCs of OGTT are shown in Table 2(Tab. 2). These ratios were highly correlated with 2-hour blood glucose levels and tAUC of OGTT. The 1-deoxySA/C18SAdiene ratio was highly correlated with 2-hour blood glucose load (r=0.700) and tAUC (r=0.569) of OGTT. Similarly the 1-deoxySO/C18SAdiene ratio was also significantly correlated with 2-hour plasma glucose level (r= 0.518) and tAUC (r=0.432) of OGTT. The scatter plots of 1-deoxySA/C18SAdiene and 1-deoxySO/C18SAdiene vs 2-hour blood glucose and tAUC of OGTT are presented in Figure 3(Fig. 3). 

**Figure 4:**
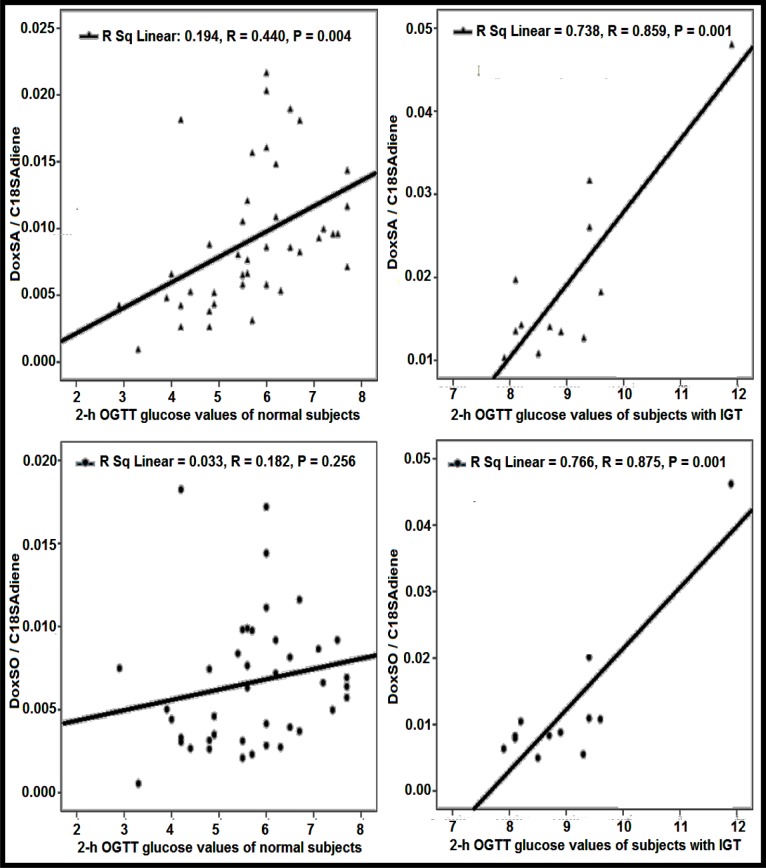
Comparison of the ratios of doxSA/C18SAdiene and doxSO/C18SAdiene against 2-h glucose values of OGTT in normal (N=41) and subjects with gestational diabetes (N=12).

The ratios of 1-deoxySA and 1-deoxySO with respect to C18SAdiene were compared between subjects with gestational diabetes and subjects having normal 2-hour glucose values of OGTT. The Pearson's correlations of 1-deoxySA/C18SAdiene and 1-deoxySO/C18SAdiene ratios with 2-h glucose values of OGTT in normal and subjects with IGT were compared. The correlations of 1-deoxySA/C18SAdiene and 1-deoxySO/ C18SAdiene vs 2-h glucose values of OGTT are r=0.859, and r=0.875 in subjects with IGT and r=0.44, and r=0.182 in normal subjects, respectively, as shown in Figure 4(Fig. 4). 

## Discussion

Oral glucose tolerance test (OGTT) is the screening test that is applied to measure plasma glucose level of an individual after ingestion of 75 grams of glucose solution. It is commonly used to define glucose tolerance for practical purposes and confirms the diagnosis of gestational diabetes, type 2 diabetes, and other metabolic diseases (Dedík et al., 2003[6]). In clinical practice simple summary measures like fasting, one-hour (1-h), two-hour (2-h) values, and area under the curve (AUC) are commonly applied to get information about the glucose tolerance (Yogev et al., 2004[28]; Siegmund et al., 2008[20]). In comparison with fasting, or 2-h glucose value AUC is the better measure to capture the essential temporal information of OGTT glucose curves. The major shortcoming of OGTT is the limitation of measuring only glucose as metabolic factor. The correlation of OGTT with another metabolic marker other than glucose at the same time will not only increase its understanding but will also increase its accuracy and precision in the diagnosis of metabolic disorders. The aim of our study is to identify the other metabolic biomarker that shows relationship with glucose during OGTT which improves the diagnostic utility of the OGTT and enhances our understanding of metabolism in health and disease. In this study glucose response vs time is not only evaluated by one-hour (1-h), two-hour (2-h), three-hour (3-h) values of OGTT, but also the total glucose response vs time was evaluated by area under the curve (AUC). The sphingoid base profile was correlated with blood glucose levels and AUCs of OGTT. The ratios of 1-deoxySA/C18SAdiene and 1-deoxySO/ C18SAdiene were also correlated with blood glucose levels and AUCs of OGTT.

Among the studied sphingoid bases the 1-deoxy-sphingoid-bases (1-deoxySA and 1-deoxySO) showed significant positive correlation with the different time points glucose level and tAUC, pAUC, and iAUC of OGTT while the C18SAdiene, C18SO, C17SO, and C16SO sphingoid bases showed significantly negative correlation. The correlations of other sphingoid bases were non-significant. The 1-deoxySA has shown strong correlation with glucose values of 1-hour, 2-hour, and 3-hour of OGTT as well with tAUC, pAUC, and iAUC; however 1-deoxySO was significantly correlated only with 1-hour, 2-hour glucose values and tAUC of OGTT. The 2-hour glucose was strongly correlated with 1-doxySA and 1-doxySO as compared to 1-hour and 3-hour correlations. Similarly the tAUC was highly correlated with 1-deoxySA and 1-doxySO in comparison with pAUC, and iAUC correlations. It has been observed that the serine-based sphingoid bases were negatively correlated while alanine-based sphingoid bases were positively correlated with glucose load of OGTT. The ratios of 1-deoxySA/C18SAdiene and 1-deoxySO/C18SAdiene with respect to blood glucose levels and AUCs of OGTT have shown significant correlation indicating that serine based sphingoid bases decreased while alanine based sphingoid bases increased with glucose load of OGTT. The C16 and C18-sphingoid bases are functionally linked to serine whereas 1-deoxySLs are linked to alanine metabolism. As the alanine to serine ratio might increase with the glucose value of OGTT therefore the ratios of 1-deoxySA/C18SAdiene and 1-deoxySO/ C18SAdiene with respect to blood glucose levels and AUCs of OGTT were highly correlated. The ratios of 1-deoxySA and 1-deoxySO with respect to C18SAdiene are higher in subjects with gestational diabetes in comparison with subjects having normal 2-hour glucose values of OGTT. This shows that alanine to serine ratio was increased in patients with gestational diabetes as compared to normal subjects. The possible mechanism might be the increased availability of intracellular alanine in the hyperglycemic conditions during OGTT. Sphingolipid metabolism represents a metabolic cross point which interconnects lipid (acyl-CoA) and amino acid (serine, alanine, and glycine) metabolism. The cellular production of serine and alanine is functionally connected to carbohydrate metabolism due to formation of their precursors 3-phosphoglycerate and pyruvate and thereby indirectly linked to carbohydrate metabolism. That is why the observed changes show a functional interaction between sphingolipids and glucose metabolism thereby increase the production of 1-deoxySLs in diabetes (Bertea et al., 2010[4]). In previous studies hyperglycemia was linked to changes of some sphingoid bases especially to the increased production of DSBs (Bertea et al., 2010[4]; Othman et al., 2012[15]). Hyperglycemic conditions are associated with elevated hepatic glucose levels and an increased glycolytic flux hence increases the formation of pyruvate and its conversion to either lactate or alanine. Elevated glucose levels could increase hepatic alanine and thereby deoxy-sphingolipids production. These results indicate a functional interaction between sphingolipid and glucose metabolism (Othman et al., 2012[15]).

This study is a valuable addition to other reported metabolomics studies on OGTT (Shaham et al., 2008[19]; Zhao et al., 2009[29]; Spégel et al., 2010[21]). In previous studies it has been reported that 1-deoxySLs are elevated in MetS (Othman et al., 2012[15]), T2DM (Bertea et al., 2010[4]), and in patients with IFG (Othman et al., 2015[14]) and their plasma levels are significantly and independently associated with a higher risk of developing (Othman et al., 2015[14]). In this study the different time points glucose level as well AUCs of OGTT were correlated with the sphingoid bases of the plasma for the first time to investigate the association of these sphingoid bases with OGTT. Similarly the ratios of 1-deoxySA/C18SAdiene and 1-deoxySO/C18SAdiene were also correlated with blood glucose level and AUCs of OGTT to investigate their association as biomarkers of diabetes. Although traditional risk factors like fasting glucose, BMI and dietary patterns inform us about the future diabetes risk, however, it will be important to know the mechanisms by which 1-deoxySLs might promote the diabetes. The correlation of 1-dexoySLs with glucose levels of OGTT revealed that these are the reliable biomarkers of OGTT. 1-deoxySLs are not only elevated early in the course of diabetes development but might also be involved in the pathogenesis of T2DM. The elevated level of 1-deoxySLs may not only serve as diagnostic biomarker but might also improve risk prediction and enable the timely prevention of diabetes (Othman et al., 2012[15]). However further studies might be required to understand the interaction between sphingolipids, and glucose metabolism. These metabolomics studies in connection with oral glucose metabolism in healthy subjects during an OGTT have the potential to advance our knowledge of metabolism in normal conditions. Moreover information about the selected sphingolipids analyzed by such approaches might improve the investigation of metabolism in health and disease. The supposed biomarkers i.e., 1-deoxySLs and their ratios with respect to C18SAdiene might be used for the identification of subjects with risk of perturbed glycemic and metabolic control and also for those subjects who require future prevention and treatment. However further investigations are required to link these metabolites with glucose level of different time points during an OGTT in normal as well as in subjects with impaired glucose tolerance. 

## Conclusion

The OGTT in normal subjects was carried out for the evaluation of impaired glucose tolerance, and risk of future diabetes in these subjects. The total blood glucose response vs time was measured by tAUC, iAUC, and pAUC. The correlation studies of AUC and different glucose levels measured during OGTT concluded that tAUC showed best correlation with the 2-hour glucose level of OGTT. The total glucose response can be better represented by the tAUC rather than iAUC, pAUC and it can be applied for the assessment of total glucose load and diabetes diagnosis. Furthermore in this study the sphingoid-base profile was explored and correlated with tAUC and 2-hour glucose level of OGTT. Our findings suggest that 1-deoxySLs and their ratios with C18SAdiene could be significantly correlated with 2-hour glucose level and tAUC of OGTT. Moreover these ratios are higher in subjects with IGT and were highly correlated with 2-hour blood glucose level of OGTT in comparison with normal subjects. The 1-deoxySLs and their ratios with C18SAdiene might be used as a biomarker for glucose metabolism and diabetes diagnosis in future and the accuracy of the OGTT for the prediction of diabetes would be much more improved by using these additional biomarkers. Our study is different from the previous studies. We explored not only the relationship between different time points blood glucose level of OGTT with total, incremental, and positive incremental AUC, to find the best method for measuring total glucose load but also correlated sphingoid-base profile to 2-hour glucose and tAUC of OGTT in order to find other novel predictors for the diagnosis of diabetes. Finding other reliable predictors along with 2-hour glucose values of OGTT is essential to enhance the predictability of diabetes and its diagnosis can be performed more accurately in the future. This might also be critical for identifying persons at high risk, and then describing this group for intervention and other preventive measures. Although traditional risk factors like fasting glucose, BMI and dietary patterns inform us about the future diabetes risk however it will be important to know the mechanisms by which 1-deoxySLs might promote the diabetes.

## Figures and Tables

**Table 1:**
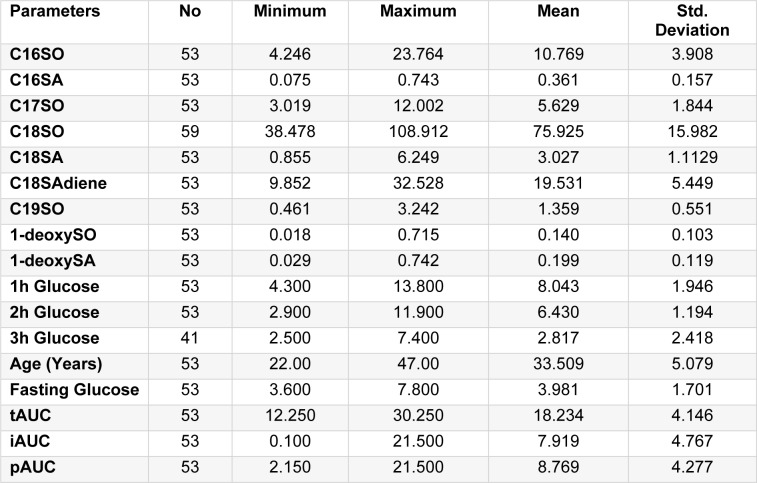
Descriptive statistics of the glucose level measured at 0, 1, 2, and 3-hour and different sphingoid-bases of participants during Oral Glucose Tolerance Test (OGTT)

**Table 2:**
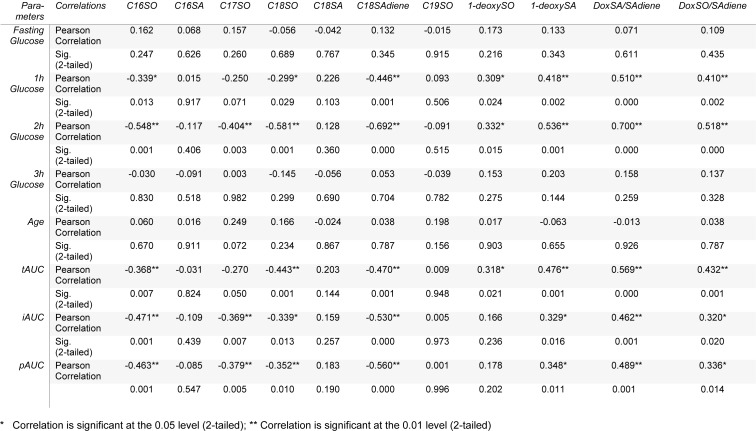
Pearson's Correlations showing the relationship of different sphingoid bases with glucose level of 0, 1, 2, and 3-hour and Total Area Under the Curve (tAUC), Incremental Area Under the Curve (iAUC), and Positive Incremental Area Under the Curve (pAUC) during an Oral Glucose Tolerance Test (OGTT)
